# Supplementary material for: Leadership – not followership – determines performance in ant teams
Source: Commun Biol. 2021 May 6;4:535. doi: 10.1038/s42003-021-02048-7 (PMC8102589; doi:10.1038/s42003-021-02048-7)
Supplement: Supplementary file 1 — Supplementary Information [file 42003_2021_2048_MOESM1_ESM.pdf]

Leadership – not followership – determines performance in ant teams: Supplementary Information

Thomas O. Richardson*[1,2], Andrea Coti[1], Nathalie Stroeymeyt†*[1,2], and Laurent Keller†[1]

[1]*Department of Ecology and Evolution, Biophore, University of Lausanne, 1015 Lausanne, Switzerland*
[2]*School of Biological Sciences, University of Bristol, Bristol Life Sciences Building, 24 Tyndall Avenue, Bristol, BS8 1TQ. United Kingdom*

† These authors contributed equally to this work.

Colony collection & maintenance

The twelve colonies used in this study all contained a single queen, around 90 workers and a complement of eggs, larvae and brood (Table. 1). The four colonies that were used in each replicate were collected one week prior to the first emigration within a given replicate. Colonies were maintained under controlled environmental conditions (50% humidity, 24-25°C, 12:12 light-dark cycle). At all times other than during the emigrations, colonies had access to inside glass tubes containing water and closed with cotton. Every seven days all colonies were fed with a mixture containing honey, eggs, agar, water and vitamins[1], *Drosophila* flies and 10% honey solution.

		Colony size (N workers)	
**Replicate**	**Collected**	**Mean±S.D.**	**Range**
1	20.06.2018	92±19	71 - 116
2	07.09.2018	86±9	75 - 95
3	12.10.2018	92±11	79 - 104

Table S1: **Colony demographics by replicate.** Each replicate consisted of four queenright colonies.

Applying unique paint codes to ant workers

To apply the paint codes, each ant was briefly anaesthetised by exposure to carbon dioxide using a FlyStuff Flypad placed under a dissecting microscope. Paint marks were applied to the anaesthetised ant using a fine entomological pin. Each ant was subjected to no more than one minute in the carbon dioxide bath. After painting, the ant was left in open air for approximately 3 minutes to recover and dry. Each newly-painted ant was photographed with a hand-held digital microscope (MAOZUA, 5MP USB Microscope). These photographs were later used to distinguish between tandem running ants that had lost multiple paint marks using the distinctive shapes of individual paint marks.

*Corresponding authors; nathalie.stroeymeyt@gmail.com

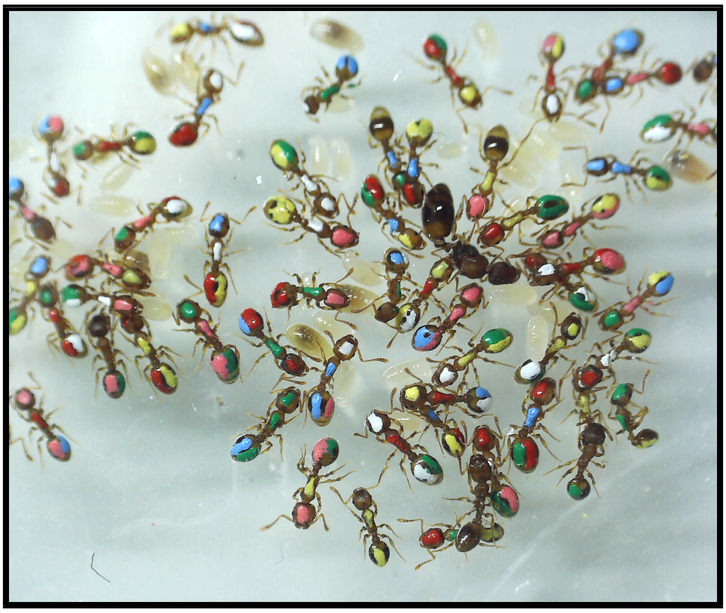

Figure S1: **Paint-marked *Temnothorax nylanderi* worker ants.** The paint marking scheme was designed to generate a set of codes that were maximally redundant, hence identification was possible even after the loss of up to two paint marks. The queen (the larger ant, visible in the centre) was not paint marked.

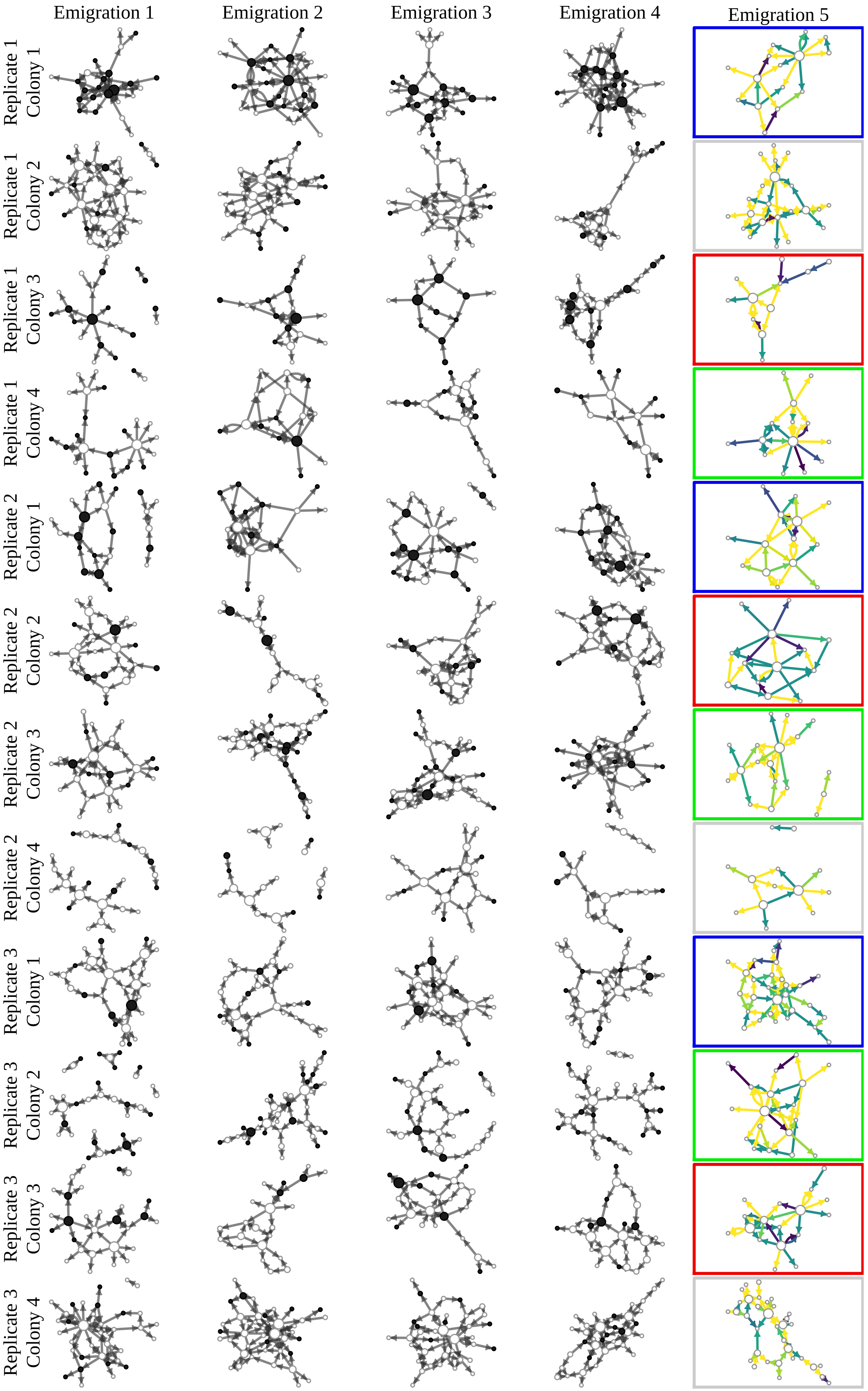
Emigration 1
Emigration 2
Emigration 3
Emigration 4
Emigration 5
Replicate 1 Colony 1
Replicate 1 Colony 2
Replicate 1 Colony 3
Replicate 1 Colony 4
Replicate 2 Colony 1
Replicate 2 Colony 2
Replicate 2 Colony 3
Replicate 2 Colony 4
Replicate 3 Colony 1
Replicate 3 Colony 2
Replicate 3 Colony 3
Replicate 3 Colony 4

Figure S2: (*Overleaf*) **Tandem recruitment networks for all 12 colonies.** Nodes represent ants. Links represent tandem runs, and are directed from the leader to the follower. Node size indicates the number of tandem runs led. Ants that were removed between the fourth and fifth emigration are coloured black. In the fifth emigration, edge colours indicate the straight-line distance. The colour of the box around the networks for the fifth emigration indicates the targeted removal treatment; grey - positive control, blue - leader & follower removal, red - leader removal, green - follower removal.

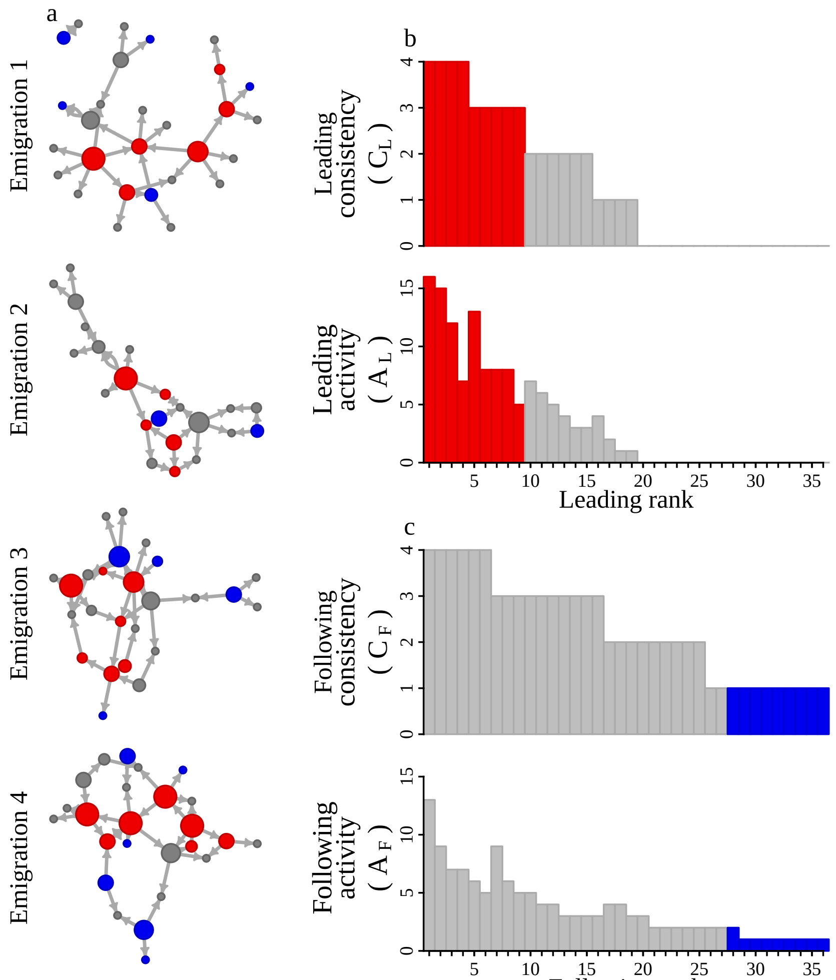

Figure S3: **Targeted removal procedure for the 'leader removal' treatment. (a)** The four baseline tandem recruitment networks for an example colony. Nodes represent ants. Coloured nodes indicate individuals that were removed after the fourth emigration. Red nodes; top-ranked leaders. Blue nodes; bottom-ranked followers. Node size indicates the number of tandem runs led. Links represent tandem runs, and are directed from the leader to the follower. **(b)** Leading ranks. Individuals were first ranked by the number of emigrations in which they led, C_L. Ties were broken by ranking according to activity in leading, A_L.Red bars; top 25 % highest-ranked leaders, which were removed from the colony after the fourth emigration. **(c)** Following ranks. Blue bars; bottom 25 % lowest-ranked followers, which were removed from the colony.

Temporal consistency of leading and following

To assess the consistency of individuals' leading and following activity, we performed a time-lagged correlation analysis to test whether the number of tandem runs an individual led (followed) in one emigration, predicts the number it led (followed) in a later emigration. As each colony underwent four baseline emigrations each separated by seven days, the pairwise correlations were measured at three different time-lags (lag = 8,16 & 24 days). These correlations should cluster around zero if individual behaviour is not consistent across emigrations.

We found that whilst the temporal consistency in the number of tandems led exhibited a clear positive bias for all time-lags (i.e., up to 24 days, Fig. S4a,d), the consistency in the number of tandems followed was considerably lower, but still slightly above chance levels (Fig. S4b,e). We also found a negative correlation between the number of tandem runs an individual followed in one emigration, and the number it led in a subsequent emigration (Fig. S4c,f)

Therefore, individual ants tend to repeatedly occupy similar topological positions within tandem recruitment networks across multiple emigrations, and hence individuals that concentrate upon leading over multiple emigrations tend to exhibit low levels of following.

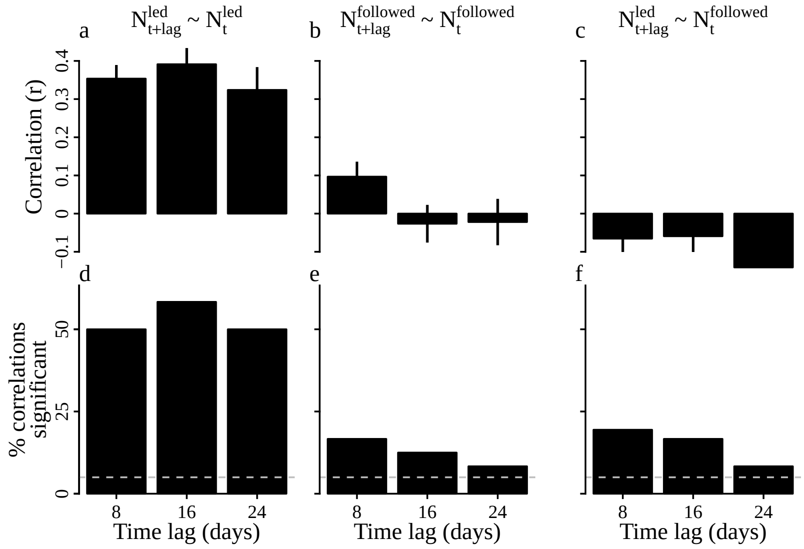

Figure S4: **Strong persistence in leading activity over multiple emigrations. (a-c)** Time-lagged correlation analysis of the number of tandem runs led and followed across multiple emigrations. Bars show the mean & standard errors of the Pearson correlation coefficients for all emigration combinations separated by a given time-lag. Note the last column shows the time-lag correlation between tasks. **(d-f)** The proportion of emigration combinations that exhibited a statistically significant correlation (two-tailed test). Dashed horizontal lines indicate the 5% threshold for statistical significance.

Testing for assortative matching between leaders and followers

In this section we describe the statistical procedures used to identify the 'matching' between consistent leaders and consistent followers within tandem runs. Following Richardson et al.[2], our first step was to calculate the joint distribution of the leading and following consistency $f^{ant}(\mathrm{C_L}, \mathrm{C_F})$, from all ants from all 12 colonies that were ever seen to tandem run during the baseline emigrations (Fig. S5a). To quantify consistency variation within tandem runs, we calculated the differences in consistency between both participants within tandem pairs. Thus for the leading task we calculated $\Delta\mathrm{C_L} = \mathrm{C_L}(\mathrm{Ldr}) - \mathrm{C_L}(\mathrm{Foll})$, where the parentheses indicate respectively the leader and follower in a given tandem run. The difference in following consistency between the leader and the follower in each tandem run, $\Delta\mathrm{C_F}$, was calculated in the same way.

Calculating these within-tandem differences revealed that most tandem runs exhibited positive $\Delta\mathrm{C_L}$ and positive $\Delta\mathrm{C_F}$ values (Fig. S5b). In other words, most tandem runs were composed of a leader that had a higher leading than following consistency, and a follower that had a higher following than leading consistency.

To assess whether this bias towards positive values of $\Delta\mathrm{C_L}$ and $\Delta\mathrm{C_F}$ was a real phenomenon, we compared the observed distribution of leader-follower consistency differences, $f^{tand}_{Obs}(\Delta\mathrm{C_L}, \Delta\mathrm{C_F})$, with that expected if leaders and followers assort at random, $f^{tand}_{Exp}(\Delta\mathrm{C_L}, \Delta\mathrm{C_F})$. To construct the expected distributions of leader-follower consistency differences, we randomly sampled tandem pairs from the joint consistency distribution, $f^{ant}(\mathrm{C_L}, \mathrm{C_F})$, and then calculated the consistency differences between the leader and the follower in each pair. This expected distribution is shown in Figure S5c.

A statistical comparison between the observed and expected distributions confirmed that the observed distribution was significantly different from the expectation (Chi-square goodness-of-fit test on 1807 tandem runs from emigrations 1-4; d.f. = 78, $\chi^2 = 2714$, $p < 0.0001$). Visualising the signed differences between the observed and expected distributions ($f^{tand}_{O-E}(\Delta\mathrm{C_L}, \Delta\mathrm{C_F})$, Fig. S5d) confirmed that the observed distribution contained an over-abundance of tandem runs in which the tandem leader was a more consistent leader than the follower, and the follower a more consistent follower than the leader. Therefore, just as during tandem running in *T. albipennis*[2], pairs of tandem running *T. nylanderi* ants are not randomly assembled, but rather their composition is consistent with a division of labour between consistent leaders in the leading role and consistent followers in the following role.

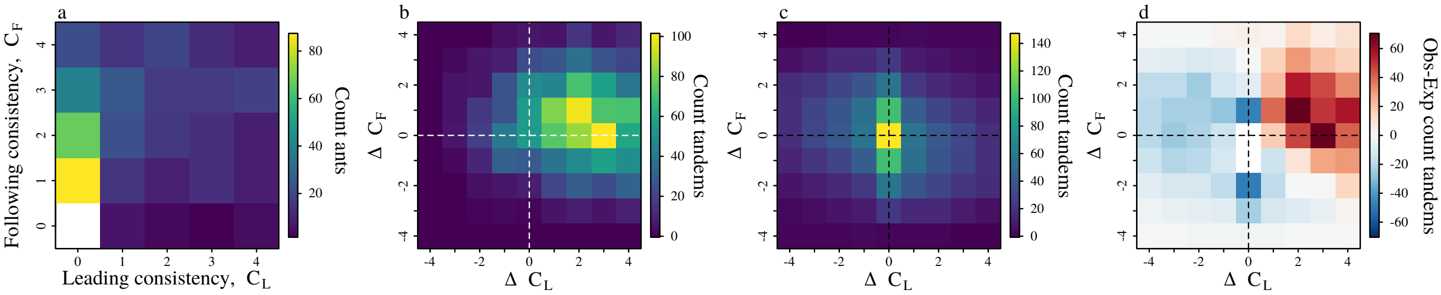

Figure S5: **Assortative matching between tandem leaders and followers. (a)** Joint frequency distribution of the leading and following consistency, $f^{ant}(\mathrm{C_L}, \mathrm{C_F})$. Each cell gives the count of the number of ants that respectively led and followed once in $\mathrm{C_L}$ and $\mathrm{C_F}$ emigrations. **(b)** Joint frequency distribution of the *differences* in leading and following consistency for the leader and follower ant in every tandem pair, $f^{tand}_{Obs}(\Delta\mathrm{C_L}, \Delta\mathrm{C_F})$. **(c)** Specialisation differences expected when $\mathrm{C_L}$ and $\mathrm{C_F}$ are independent, $f^{tand}_{Exp}(\Delta\mathrm{C_L}, \Delta\mathrm{C_F})$. **(d)** Signed difference between the observed and expected joint distributions, $f^{tand}_{O-E}(\Delta\mathrm{C_L}, \Delta\mathrm{C_F})$. This distribution is skewed towards the upper right quadrat were the tandem leader is a more consistent leader than the tandem follower, and where the follower is a more consistent follower than the leader.

Permutation tests for task switching

As it may be possible that ants with a low C_L may *by definition* have low P(F→L) because they led in few emigrations, we performed data permutations in which the identities of the tandem leaders (respectively followers) were randomly reshuffled within each emigration. Shuffling the identities of the leaders and followers within each emigration disrupted the association between consistency and particular tandem running sequences. However, the procedure also preserved both the C_L and C_F of each individual (since an ant that was involved as a leader or a follower in a given emigration in the original data still had that role in the permuted data), and also preserved the integrity of tandem running sequences within each emigration (e.g. a particular tandem running sequence of F,F,L,L F,L in the original data was found unchanged in the permuted data, albeit assigned to a different leader). By constraining the permutations to disrupt only the putative associations between consistency and switching, we were thus able to test whether the observed associations were real phenomena or mere statistical artefacts.

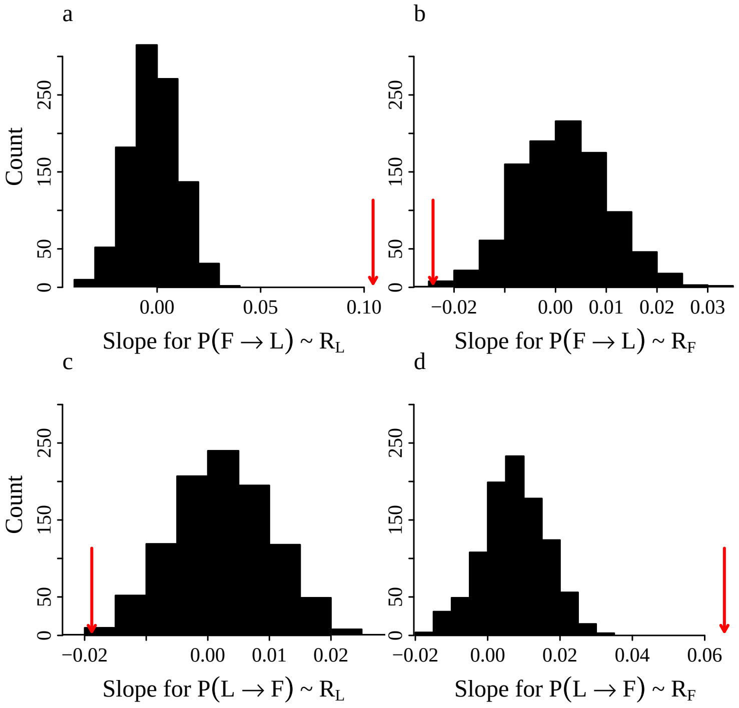

Figure S6: **Permutation tests confirm the presence of asymmetric task switching.** Panels show the observed and expected per-tandem task switching probability, for the following to leading transitions **(a-b)**, and for the leading to following transition **(c-d)**. Histograms show slopes from 1000 data permutations where individual tandem running sequences were decoupled from individual consistency. Red arrows indicate the observed slope, corresponding to the fits shown in Figure 2 in the main text. The two-tailed probabilities that the observed value is drawn from the expected distribution were $p<0.001$ for panel (a); $p = 0.006$ for (b); $p=0.024$ for (c); and $p < 0.001$ for (d).

Targeted removals modify tandem pair composition

The targeted removal treatments succeeded in modifying the tandem pair composition. The removal of prominent leaders (red bars) resulted in tandem runs in which the leaders were both less consistent and less active in leading than tandem runs in the baseline emigrations (red versus black bars in Fig. S7 a,c,e), and than tandem runs in the positive control (red versus grey bars in Fig. S7 a,c,e).

The simultaneous removal of prominent leaders and prominent followers resulted in tandem runs in which the leaders were less consistent and less active in leading than tandem runs in the baseline emigrations (blue versus black bars in Fig. S7 a,c,e), and than tandem runs in the positive control (blue versus grey bars in Fig. S7 a,c,e). This removal treatment also resulted in tandem runs in which the followers were less consistent and less active in following than tandem runs in the baseline emigrations (blue versus black bars in Fig. S7 b,d,f).

The removal of prominent followers resulted in tandem runs in which the followers were less consistent and less active in following than tandem runs in the baseline emigrations (green versus black bars in Fig. S7 b,d,f). However, the consistency and activity of the tandem runs in this removal treatment were not different to those in the positive control (green versus grey bars in Fig. S7 b,d,f).

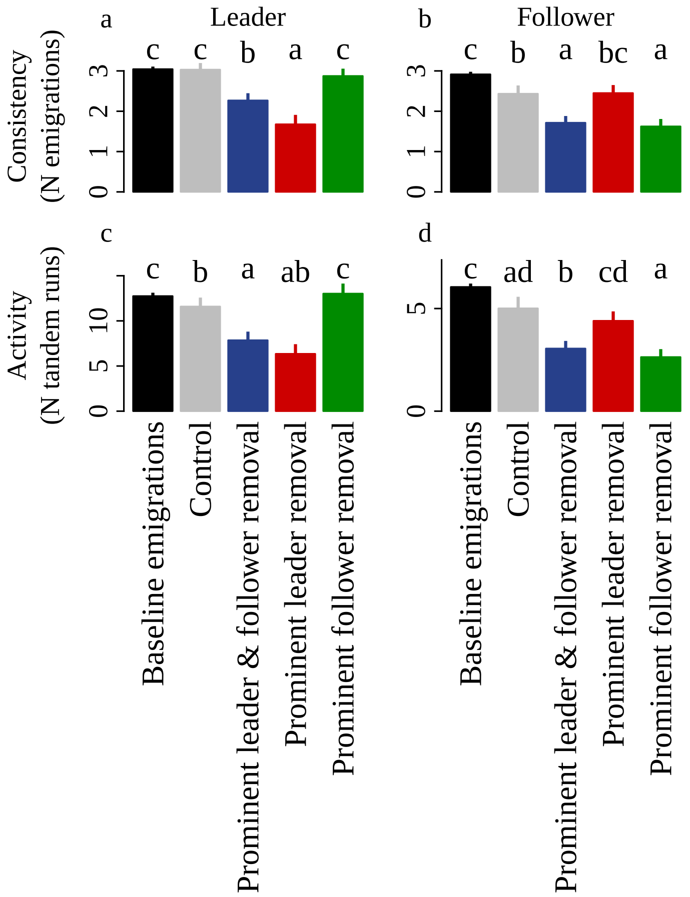

Figure S7: **Manipulating tandem pair composition by targeted worker removals.** Bars & errors represent the mean & standard error, where the averages are calculated across the colony means. Letters indicate results from post-hoc contrasts (one LMM for each panel, with treatment as the main effect, and colony as a random effect). Plots & statistics based on all 2143 tandem runs.

Multimodel inference and model averaging

The first step in the multi-model inference procedure was to construct one mixed model for each of the predictor combinations, and then to measure the parsimony of each model. To quantify model parsimony we used the small sample size correction of the Akaike Information Criterion (AICc,[3]) as this is recommended when the ratio of the sample size n, to the number of fitted parameters k, is less than 40 (here $n = 135$ tandem runs & $k = 7$ for the most complex model, hence $n/k = 19$). The next step was to calculate the AICc differences Δ_i between each model i and the most parsimonious model, from which we obtained the Akaike weight w_i for each model i, that is,

$$w_i = \frac{\exp(-\frac{1}{2}\Delta_i)}{\sum\limits_{i \in I} \exp(-\frac{1}{2}\Delta_i)}$$

where I represents the ensemble of models tested. The Akaike weight for a given model represents the probability that model i is the model that best approximates the truth, given the data and the set of models being compared[4].

The last step in the model selection procedure was to identify the 95% 'confidence set' $I_{95\%}$ by calculating the cumulative sum of the Akaike weights from the top-ranked (i.e. most parsimonious) model downwards until the accumulated Akaike weight reached 0.95. As the Akaike weights can be interpreted as probabilities, it is 95% certain that the confidence set contains the 'true' model.

To identify the most important determinants of tandem progress, for each predictor we summed the Akaike weights across all models in the confidence set in which that predictor appeared. Hence a predictor that appeared in all models or many of the high-ranked models would receive a summed Akaike weight ('relative importance') close to 1, whereas a predictor that appeared in the least likely models would have a relative importance close to 0[5,6].

Finally, to estimate the effect sizes associated with each predictor, we performed model averaging across all the candidate models in the confidence set. This involves calculating a weighted average coefficient for each parameter across all of the confidence set models containing said parameter;

$$\beta = \frac{\sum\limits_{i \in I_{95\%}} w_i \beta_i}{\sum\limits_{i \in I_{95\%}} w_i}$$

where β_i represents the coefficient for a given parameter in model i, and w_i represents the Akaike weight for model i. As in our analysis the evidence in favour of the top-ranked model was equivocal, we performed 'full model averaging'[4,7]. In this variant of model averaging, all models in which a given parameter does not appear contribute a value of 0 to the weighted average, hence parameters that appear in only a few models in the confidence set, or that appear in only low-ranked models, will receive a low coefficient.

All mixed effects models were implemented using the package *lme4* version 1.1-13 for R[8]. All multi-model inference and model averaging calculations were carried out using the package *MuMIn* version 1.40.0 for R[9].

i	**Response**	**Candidate model**	**d.f.**	**AICc**	Δ_i	w_i	**Acc'** w_i	**ER**
1	Success rate	C_L	8	310.4	0	0.105	0.1	1
2		$C_F + C_L + C_F{:}C_L$	10	310.6	0.3	0.0916	0.187	1.15
3		$A_L + C_L$	9	311.2	0.8	0.0694	0.253	1.52
4		$A_L + C_F + C_L + C_F{:}C_L$	11	311.3	1	0.0647	0.314	1.63
5		$A_F + C_L$	9	311.8	1.5	0.0509	0.363	2.07
6		$C_F + C_L$	9	311.8	1.5	0.0508	0.411	2.08
7		C_L + Rank	9	312.1	1.8	0.0431	0.452	2.44
8			7	312.3	2	0.0391	0.489	2.69
9		$A_F + A_L + C_L$	10	312.8	2.4	0.0315	0.519	3.35
10		$A_L + C_F + C_L$	10	312.8	2.4	0.0313	0.549	3.36
...								
35		$A_F + C_F$	9	315.6	5.2	0.00778	0.949	13.6
1	Straight-line distance	C_L	9	715	0	0.251	0.237	1
2		$C_F + C_L$	10	716.1	1	0.149	0.378	1.68
3		$A_F + C_F + C_L$	11	717	2	0.0912	0.464	2.75
4		$C_F + C_L + C_F{:}C_L$	11	717.6	2.6	0.0679	0.528	3.69
5		$A_F + C_L$	10	717.8	2.8	0.0617	0.586	4.06
6			8	717.9	2.8	0.0603	0.643	4.16
7		C_F	9	718.4	3.4	0.0461	0.687	5.43
8		$A_L + C_L$	10	718.5	3.5	0.0431	0.728	5.81
9		$A_F + C_F + C_L + C_F{:}C_L$	12	718.8	3.7	0.0389	0.764	6.44
10		$A_F + C_F$	10	719.3	4.2	0.03	0.793	8.35
...								
21		$A_F + A_L + C_F$	11	722	7	0.00766	0.945	32.7
1	Time taken	$A_F + A_L + C_F + C_L + C_F{:}C_L$	13	1612.8	0	0.227	0.214	1
2		$A_F + A_L + C_F + C_L$ + Rank $+ C_F{:}C_L$	14	1613.1	0.3	0.196	0.398	1.16
3		$A_F + A_L + C_F + C_L + A_F{:}A_L + C_F{:}C_L$	14	1614.4	1.6	0.102	0.494	2.23
4		$A_F + A_L + C_F + C_L$ + Rank $+ A_F{:}A_L + C_F{:}C_L$	15	1614.7	1.9	0.0866	0.576	2.62
5		$A_F + C_F + C_L + C_F{:}C_L$	12	1614.8	2	0.0842	0.655	2.69
6		$A_F + C_F + C_L$ + Rank $+ C_F{:}C_L$	13	1615.1	2.2	0.0736	0.725	3.08
7		$A_L + C_F + C_L + C_F{:}C_L$	12	1616.1	3.2	0.0446	0.767	5.08
8		$A_F + A_L + C_F + C_L$	12	1616.3	3.5	0.0391	0.804	5.8
9		$A_L + C_F + C_L$ + Rank $+ C_F{:}C_L$	13	1616.3	3.5	0.039	0.84	5.8
10		$A_F + A_L + C_F + C_L$ + Rank	13	1616.6	3.8	0.0344	0.873	6.58
...								
15		$A_F + C_F + C_L$ + Rank	12	1618.5	5.7	0.0131	0.943	17.3

Table S2: **Identifying the most important predictors of tandem run performance.** A 95% confidence set was calculated for the binary success, and for the distance the tandem covers. The 95% confidence set includes all models whose cumulative Akaike weight, Acc' $w_i \leq 0.95$. ER - evidence ratio for the top-ranked model $i = 1$ over the ith model. Statistics based on forward tandem runs from emigration 5 for which both participants had participated in tandem running during the baseline emigrations.

Testing for predictor collinearity

Statistical models in which there are several correlated predictors may suffer from elevated levels of noise in the estimates of the model coefficients and their standard errors [10]. As the predictors in the multi-model analysis of tandem quality (i.e. consistency and activity) might sometimes be correlated with one another, we performed a further analysis to quantify the level of collinearity between the predictors in each candidate model.

To assess predictor collinearity we measured two quantities for every model in the 95% confidence set. The first was the absolute value of the Pearson correlation coefficient, $|r|$, which is defined for each unique pair of predictors in each model. Thus, for a model with n predictors (excluding the intercept) there are (n×n-1)/2 pairwise correlations. To identify variables that have high collinearity with other variables, for each model we calculated the mean of the n-1 correlation coefficients associated with each predictor, $|\bar{r}|$. Predictors with $|\bar{r}|$ in excess of 0.85 [11], 0.7 [10] or 0.4 [12] are typically considered to be collinear with the other predictors, and thus should be considered for exclusion from the model. The second measure of predictor collinearity was the variance-inflation factor. Predictors with VIF greater than 10 [13–15] or 5 [16,17], are typically considered to be collinear, and so candidates for exclusion. As estimation of the VIF is more complex for models that combine fixed and random effects (as here), we used the *car* pakage (version 3.0-3) for *R* to estimate the generalised variance inflation factor (gVIF, [18]).

Overall, our analysis revealed little evidence of collinearity. For example, among the models predicting

tandem run success or failure, only 1 of the 77 terms had gVIF > 5 (Fig. S8a). For the models predicting the straight-line distance, 0 of the 19 terms had gVIF > 5 (Fig. S8b).

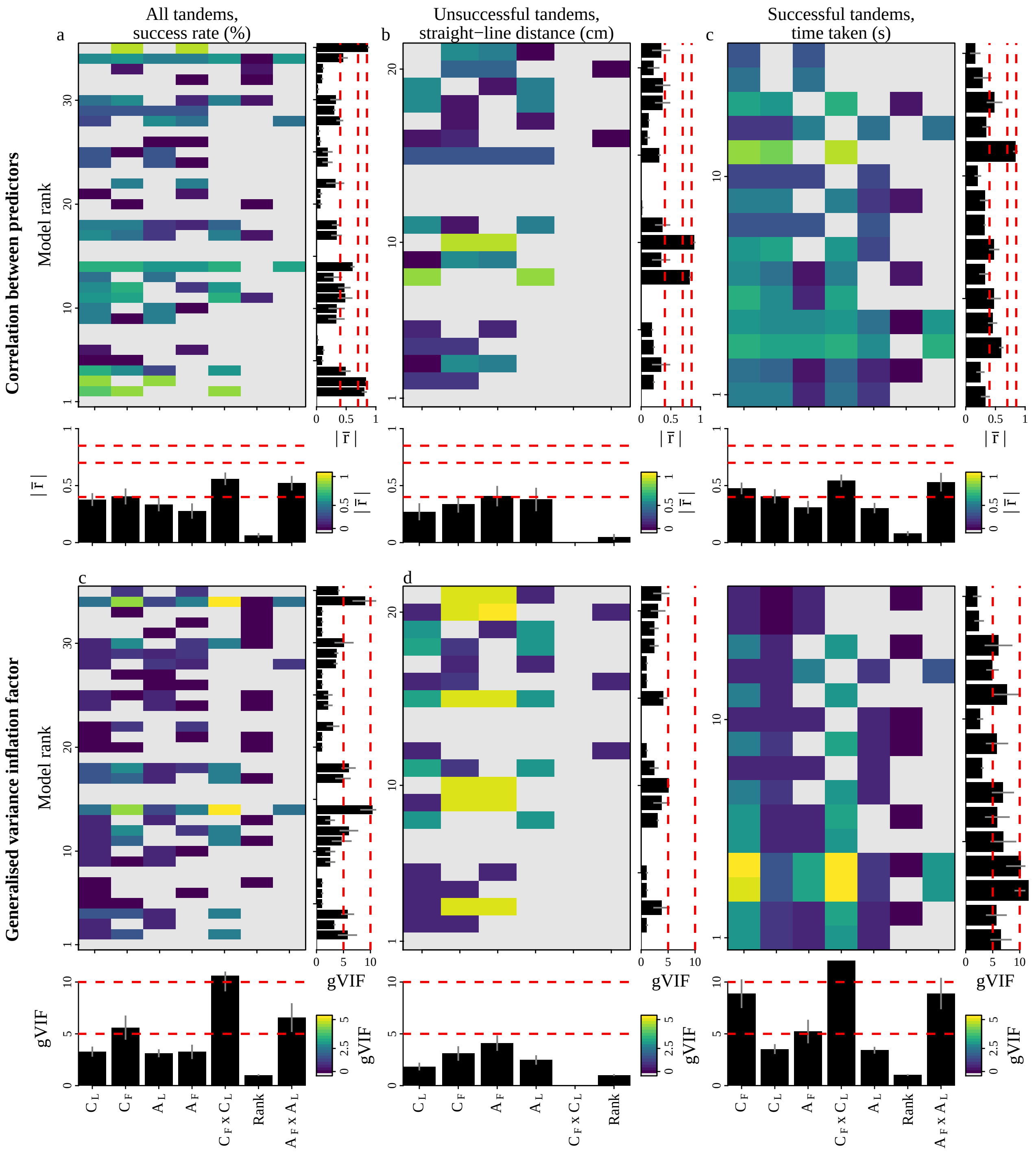

Figure S8: **Quantifying predictor collinearity in models of tandem run performance.** Upper heatmaps **(a,b)** show the mean absolute predictor correlation, $|\bar{r}|$, for each term in each model in the 95% confidence set. Lower heatmaps **(c,d)** show the VIF, for each term in each model. Grey cells indicate terms that were not in the model. Marginal boxplots show the heatmap row and column means. Bars & errors represent the mean & standard error. The bars & errors in the horizontal barplots represent the mean & standard errors within the columns of the heatmap (i.e. within predictors), whereas the vertical barplots give the same for the rows (i.e. within each model). Dashed red lines show commonly-used rejection thresholds.

Controlling for predictor collinearity

The influence of overly collinear predictors can be reduced by removing those with the highest correlations with the other predictors[11,12]. Therefore, to investigate the influence of the slightly collinear predictors identified by the mean absolute correlation, $|\bar{r}|$, we filtered the models to remove all model terms with $|\bar{r}| > 0.4$. Then, after removing duplicate models from the global set (caused by the term removal), we calculated the AIC and the Akaike weights of the simplified models, and then identified a new 95% confidence set. Finally, we used the Akaike weight for each model to calculate the importance and the model-averaged coefficient for each predictor.

As in the original analysis, the consistency of the leader in the leading role, C_L, was again the most important predictor of both the probability of success of a tandem run, and the distance that unsuccessful tandem runs travelled towards the new nest (Fig. S9a). Furthermore, averaging the coefficients across the models in these new 95% confidence sets, revealed that for both the tandem success rate and the straight-line distance the coefficient associated with C_L was positive, with lower error bound above zero (Fig. S9b).

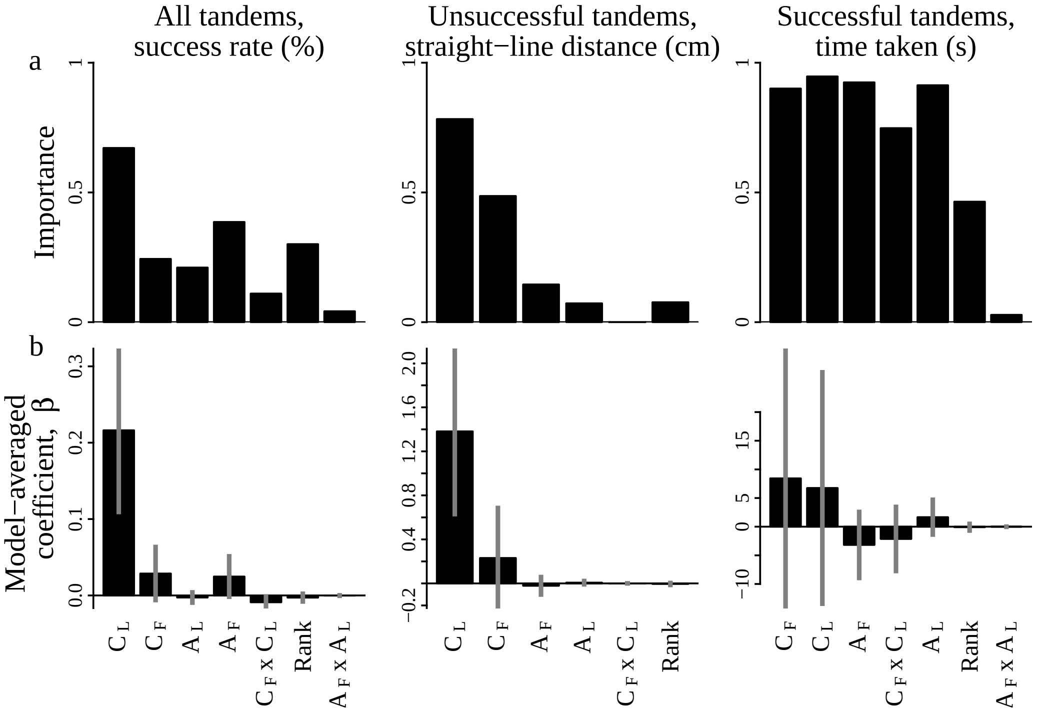

Figure S9: **Model averaging applied to models with collinear predictors removed. (a)** Relative predictor importance, and **(b)** Model-averaged coefficients across all models in the 95% confidence set. Bars & errors represent means & standard errors for each predictor; C: consistency; A: activity. Subscripts indicate the task. 'Rank' indicates the within-emigration tandem rank ordering. Statistics based on forward tandem runs from emigration 5 in which there was no follower switching, and in which both participants had engaged in tandem running during emigrations 1-4.

References

[1] Bhatkar, A. and W. Whitcomb (1970). Artificial diet for rearing various species of ants. *Florida Entomologist*, 229–232.

[2] Richardson, T. O., C. Mullon, J. A. Marshall, N. R. Franks, and T. Schlegel (2018). The influence of the few: a stable 'oligarchy' controls information flow in house-hunting ants. *Proc. R. Soc. B 285*(1872), 20172726.

[3] Sugiura, N. (1978). Further analysts of the data by Akaike's information criterion and the finite corrections. *Communications in Statistics-Theory and Methods 7*(1), 13–26.

[4] Symonds, M. R. and A. Moussalli (2011). A brief guide to model selection, multimodel inference and model averaging in behavioural ecology using Akaike's information criterion. *Behavioral Ecology and Sociobiology 65*(1), 13–21.

[5] Burnham, K. P. and D. R. Anderson (2003). *Model selection and multimodel inference: a practical information-theoretic approach.* Springer Science & Business Media.

[6] Burnham, K. P., D. R. Anderson, and K. P. Huyvaert (2011). AIC model selection and multimodel inference in behavioral ecology: some background, observations, and comparisons. *Behavioral Ecology and Sociobiology 65*(1), 23–35.

[7] Lukacs, P. M., K. P. Burnham, and D. R. Anderson (2010). Model selection bias and Freedman's paradox. *Annals of the Institute of Statistical Mathematics 62*(1), 117.

[8] Bates, D., M. Maechler, B. Bolker, and S. Walker (2013). *lme4: Linear mixed-effects models using Eigen and S4.* R package version 1.0-5.

[9] Bartoń, K. (2017). *MuMIn: Multi-Model Inference.* R package version 1.40.0.

[10] Dormann, C. F., J. Elith, S. Bacher, C. Buchmann, G. Carl, G. Carré, J. R. G. Marquéz, B. Gruber, B. Lafourcade, P. J. Leitão, et al. (2013). Collinearity: a review of methods to deal with it and a simulation study evaluating their performance. *Ecography 36*(1), 27–46.

[11] Elith, J., C. H. Graham, R. P. Anderson, M. Dudík, S. Ferrier, A. Guisan, R. J. Hijmans, F. Huettmann, J. R. Leathwick, A. Lehmann, et al. (2006). Novel methods improve prediction of species' distributions from occurrence data. *Ecography 29*(2), 129–151.

[12] Suzuki, N., D. H. Olson, and E. C. Reilly (2008). Developing landscape habitat models for rare amphibians with small geographic ranges: a case study of siskiyou mountains salamanders in the western usa. *Biodiversity and Conservation 17*(9), 2197–2218.

[13] Belsley, D. A. (1991). *Conditioning diagnostics: Collinearity and weak data in regression.* Number 519.536 B452. Wiley New York.

[14] Hair, J. F., R. E. Anderson, R. L. Tatham, and W. C. Black (1995). Multivariate data analysis new york. *NY: Macmillan.*

[15] Neter, J., M. Kutner, W. Wasserman, C. Nachtsheim, and J. Neter (2004). Applied linear regression models 4th ed.

[16] Sheather, S. (2009). *A modern approach to regression with R.* Springer Science & Business Media.

[17] James, G., D. Witten, T. Hastie, and R. Tibshirani (2013). *An introduction to statistical learning*, Volume 112. Springer.

[18] Fox, J. and G. Monette (1992). Generalized collinearity diagnostics. *Journal of the American Statistical Association 87*(417), 178–183.
